# Current status of stereotactic ablative body radiotherapy in the UK: six years of progress

**DOI:** 10.1259/bjro.20190022

**Published:** 2019-07-19

**Authors:** Gail Distefano, Satya Garikipati, Helen Grimes, Matthew Hatton

**Affiliations:** 1 Royal Surrey County Hospital, Surrey, UK; 2 CVSSP, University of Surrey, Surrey, UK; 3 Weston Park Hospital, Sheffield, UK; 4 University College London Hospitals, London, UK

## Abstract

**Objective:**

To update the 2012 UK stereotacticablative radiotherapy (SABR) Consortium survey and assess the development of SABR services across the UK over the past 6 years. Use the results to share practice and continue to drive forward technique development, aid standardization and by highlighting issues, improve access to SABR services and trials across the UK.

**Methods:**

In January 2018, an online questionnaire was sent by the UK SABR Consortium to 65 UK radiotherapy institutions covering current service provision and collecting data on immobilization, motion management, scanning protocols, target/OAR delineation, planning, image-guidance, quality assurance and future plans.

**Results:**

50 (77%) institutions responded, 38 ( *vs* 15 in 2012) indicated they had an active SABR programme with the remaining 12 centres intending to develop a SABR programme

Documented changes include the development of Linac delivered SABR to non-lung sites, an increase in centres using abdominal compression (14 *vs* 2) and the introduction of four-dimensional cone beam CBCT. Current practice is broadly in line with UK SABR Consortium and European guidelines.

**Conclusion:**

This 2018 survey shows a welcome increase in SABR provision, surpassing 2012 projections. However, it is clear that the UK SABR program needs to continue to expand to ensure that patients with oligometastatic disease have access and SABR for early stage lung cancer is available in all centres. Updated guidance that addresses variability in target delineation, image guidance and reduces patient specific quality assurance is warranted.

**Advances in knowledge:**

Documented progress of UK SABR across all treatment sites over the last six years, barriers to implementation and future plans.

## Introduction

Stereotactic ablative body radiotherapy (SABR) uses hypofractionated dose schedules (3– to 8 fractions) and high precision treatment delivery to improve local control of disease. The radiobiological rationale for hypofractionation: that delivery of a few large fractions over a short overall treatment time will achieve a greater therapeutic ratio than delivery of standard treatment regimens of 20 or more fractions, is indicated in numerous studies for a range of clinical indications.^1–5^

However, there are significant challenges associated with introducing SABR into the clinic due to the high dose fractions, the non-standard method of dose prescription and the complex nature of both planning and delivery.^6,7^

The UK SABR Consortium was established in 2008 with the aim of achieving a consensus on how best to develop, implement and research SABR in the UK. The UK SABR Consortium guidelines,^8^ which are intended to standardise UK implementation and ensure safe delivery of SABR were originally written for early stage lesions in the peripheral lung, however this guidance has been updated and significantly re-structured and covers the application of SABR for other primary tumours and oligo-metastatic disease. The most recent version is available online (https://www.sabr.org.uk/wp-content/uploads/2019/04/SABRconsortium-guidelines-2019-v6.1.0.pdf).

In the UK, the 2011 National Radiotherapy Implementation Group (NRIG) report (SBRT: Guidelines for Commissioners, Providers and Clinicians in England 2011),^9^ recommended that SABR had become a standard of care for the management of early stage medically inoperable peripheral non-small-cell lung carcinoma.

In 2012 a comprehensive survey^10^ was led by the Consortium to quantify the number of UK centres actively treating with SABR and the number intending to develop a SABR service in the next 2 years, obtain details of current practice, identify current and future clinical sites being treated with SABR, identify the equipment used in different institutions to match centres starting up, quantify the resource implications of a SABR service, determine if Consortium guidelines^8^ are being adhered to, guide best practice and alert outlying centres to possible improvements in workflow and quality.

Over the last 6 years, there have been several key developments both in terms of technology and infrastructure. These include:

1. Funding from NHS England for a mentorship scheme that supported three centres in developing and introducing a lung SABR service
2. In 2013 the NHS Commissioning Board (NHS CB) agreed to commission Stereotactic Body Radiotherapy/Stereotactic Radiosurgery for patients with early non-small cell lung cancer^11^ and cerebral metastasis.^12^
3. A national dosimetry audit for Lung SABR was developed and conducted in 24 centres.^13^ This audit is now used by the Radiotherapy Trials Quality Assurance (RTTQA) group as a pre-requisite for clinical trials
4. In 2015 NHS England’s Commissioning through Evaluation (CtE) programme^14,15^ enabled 17 centres to start treating a limited number of patients with SABR for oligometastatic disease, re-irradiation of the pelvis and spine and hepatocellular carcinoma.
5. The development of national portfolio of SABR research studies to investigate the utility of SABR in the treatment of oligometastatic disease, primary lung, prostate, pancreas and hepatobiliary malignancies.^16–20^

Therefore, it was considered timely to conduct a second survey to update results from 2012^10,21^ and see how the SABR landscape in the UK has evolved. The aim was to ascertain the progress being made in the implementation of SABR treatment, obtain details of current practice in centres with an active treatment programme and identify barriers to implementation/progress. Any issues highlighted by the survey could then be addressed within the NHS to improve access to SABR services and trials in the UK.

## Methods and materials

An online questionnaire was sent to the Consortium membership to identify a local SABR Lead at 65 UK radiotherapy centres in January 2018. The survey consisted of 72 questions and was divided into 5 sections to allow completion by different relevant staff groups (see suppl 1). Non-responders were followed up by an email to the Head of Physics in April, which increased the final response rate to 77% (50/65 UK institutions).

The questionnaire (updated since 2012, to reflect advancements in technology and current service provision) covered several areas, often in considerable detail: current and intended number of patients being treated for each clinical site; immobilization and motion management methods; CT scanning protocols; target and OAR delineation; treatment planning; image-guidance and treatment protocols; QA methods.

Amongst “treating” centres the response rate to some questions was partial due to the wide scope of the questionnaire. Results are therefore quoted as a fraction with the denominator indicating the number of the centres responding to each question. Centres that reported that they were not currently treating SABR were only included in analysis relating to questions on four-dimensional CT (4DCT) scanning experience, barriers to implementation and future plans. In contrast to 2012, data for intracranial SRS/T have been collected as one of the non-lung SABR sites for oligometastatic disease. Results from this survey have been compared to those from 2012,^10^ and to national and international guidelines/recommendations including UK SABR Consortium,^8^ ESTRO ACROP,^22^ AAPM,^23^ Australian and New Zealand^24^ and EORTC.^25,26^

## Results

### Current status and future expectations of UK SABR provision

50/65 (77%) centres responded to the questionnaire. The number of centres with an active SABR program has greatly increased since 2012, 38/50 (76%) *vs* 15/48 (31%) responding centres, with a further 12/50 responding centres planning to start SABR in the next two years. Intracranial SRS/T data were not collected in 2012.

**Figure 1.**
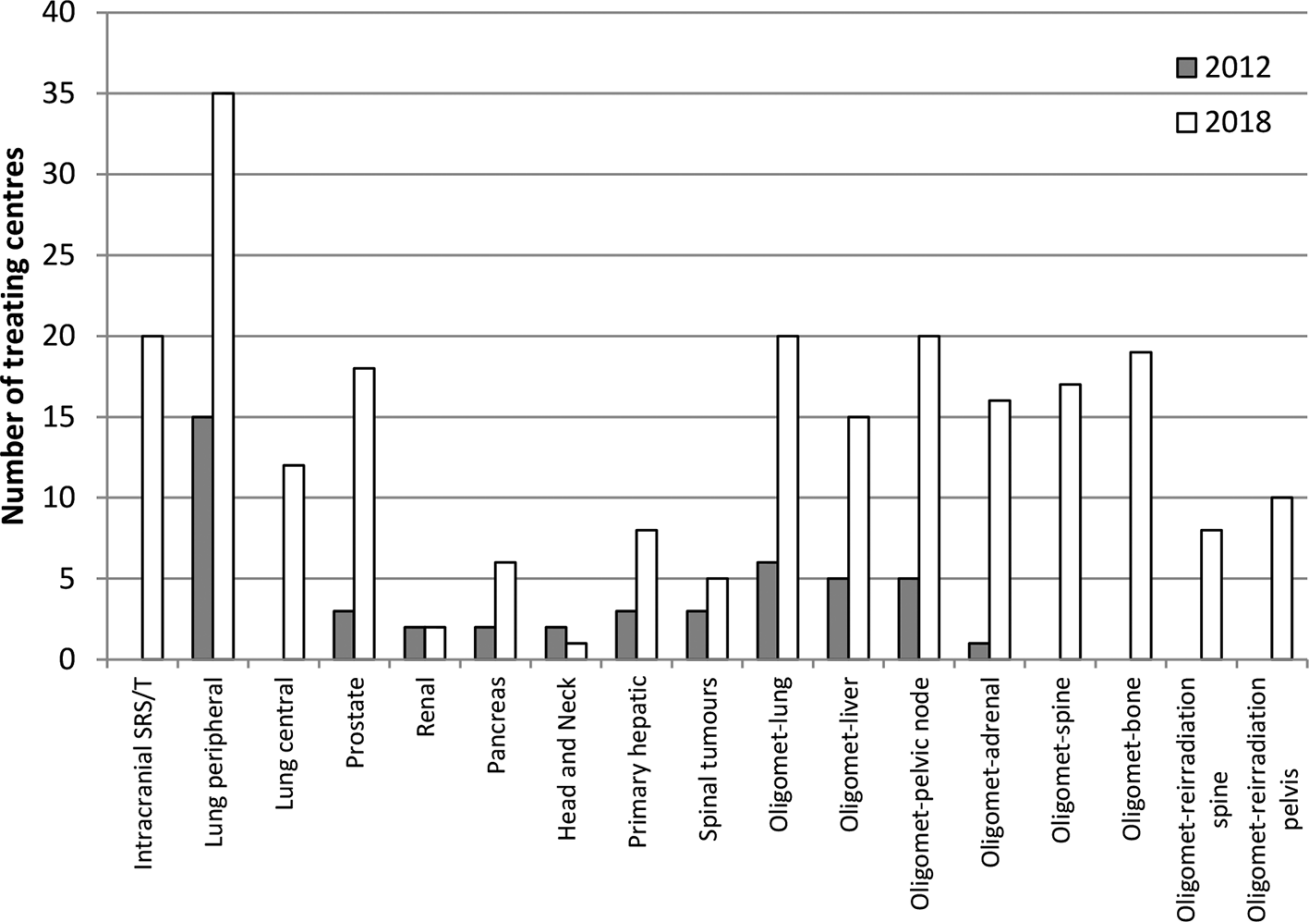
Graph showing results from the 2018 survey of the number of UK centres with an active SABR programme compared to 2012. SABR, stereotactic ablative radiotherapy.

SABR for Lung cancer—Centres treating primary peripheral lung cancers have more than doubled since 2012 (15/48–35/50, Figure 1), in line with the intentions we documented in 2012.The number of patients with peripheral non-small-cell lung carcinoma treated by each centre per annum varied from 1 to 10 to over 200 (range described in Table 1). 26/35 (74%) of responding centres are treating more than 25 patients per annum, the minimum level of provision recommended by NRIG.^9^

**Table 1:** The number of patients with peripheral NSCLC treated by each centre per annum

**No of patients treated per annum**	**No of centres** (**% of 35 treating centres**)
1–10	5 (14%)
11–25	4 (11%)
26–50	9 (25%)
51–75	7 (20%)
76–100	2 (6%)
101–150	2 (6%)
151–200	3 (9%)
200–250	3 (9%)

NSCLC, non-small-cell lung carcinoma.

The number of patients treated per annum from all centres has increased from 458 in 2012 to 1676 (Table 2). 12/35 centres are also treating central lung tumours.

**Table 2.** Annual numbers of treated patients in 2018 compared to 2012 for (a) primary sites and (b) oligometastatic indications

**Primary** **Sites**	Intracranial	Lung peripheral	Lung central	Prostate	Renal	Pancreas	Head & Neck	Liver	Spine
2012	n/a	458	n/a	31	2	19	31	4	40
2018	2176	1676	42	202	12	36	6	74	18

**Table:** 

**Oligometastases**	
2012	144
2018	984

SABR for other primary sites—30/50 (60%) responding centres deliver SABR to non-lung sites with intracranial (20 centres) and prostate (18 centres) being most common sites. Centres treating prostate and primary hepatic tumours have increased by six and two-fold respectively.

SABR for oligo-metastatic disease —27/50 (54%) responding centres are offering SABR to the range of sites required for treating oligo-metastatic disease. This has significantly increased since 2012 where the number of patients treated per annum has increased from 144 to 984 (Table 2).

### Barriers in implementing a SABR service

The response to this question allowed centres to tick multiple options/barriers (based on previous survey) with additional free text to detail specific challenges. While lack of NHS contracts continues to be a barrier for the non-SABR centres there are other challenges including lack of staff, equipment, training to existing staff, machine time on linacs, MRI capacity and lack of resources that have been highlighted as impacting on expanding SABR service in treating centres.

For peripheral lung SABR, 8/46 (17%) responding centres indicated they had problems with obtaining an NHS contract and three centres highlighted loss of an existing contract despite having the expertise. 9/42 (21%) responding centres indicated lack of staff, and 6/42 (14%) responding centres ticked lack of training to existing staff as challenges. Other challenges documented were lack of machine time on linacs (12/42 (29%) with 22/42 (52%)) of responding centres raising other issues including specific technical challenges with motion management and lack of MRI capacity.

A major concern is that for SABR to oligometastatic disease, intracranial SRS/T and implementation of/recruitment into SABR trials: lack of funding/NHS contract seems to be the major barrier as indicated by 13/19 (68%), 21/42 (50%) and 21/41 (51%) of responding centres respectively.

33/50 (66%) responding centres are currently referring to a different centre for SABR to various sites. Inequity in terms of geography has been highlighted by four centres where patients suitable for SABR refuse to travel and thereby are deprived of the service.

### Managing tumour motion and CT simulation

**Figure 2.**
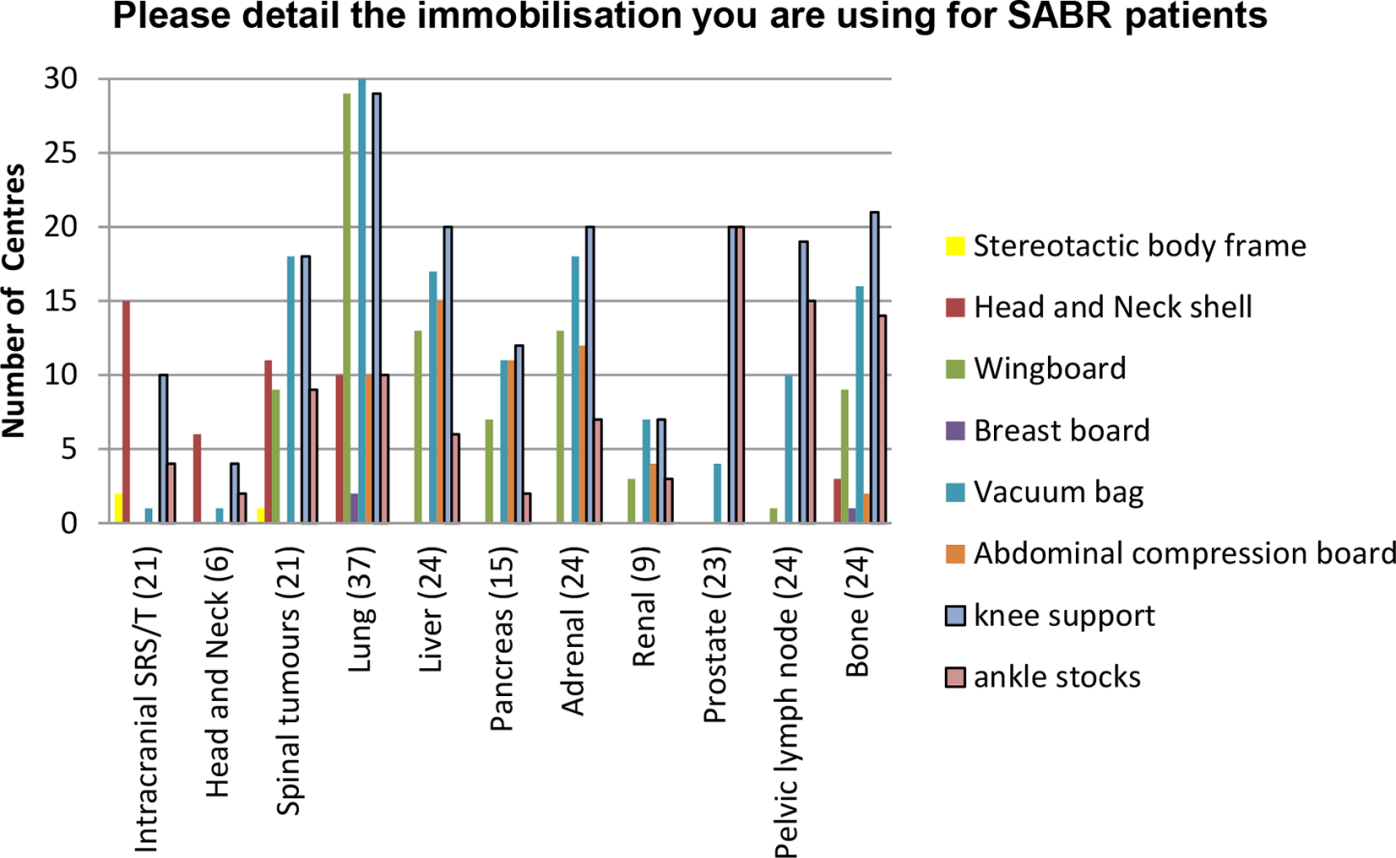
Immobilization devices currently used in the UK for SABR (38 treating centres). SABR, stereotactic ablative radiotherapy.

Immobilization and management of tumour motion is essential to accurately deliver a high dose to the target. A range of immobilization devices are used throughout the UK, as in Figure 2. In summary, it is worth noting that established practice mainly utilizes standard immobilization devices ( ±abdominal compression), with the stereotactic body frame only used by 2/37 (5%) responding centres.

**Figure 3.**
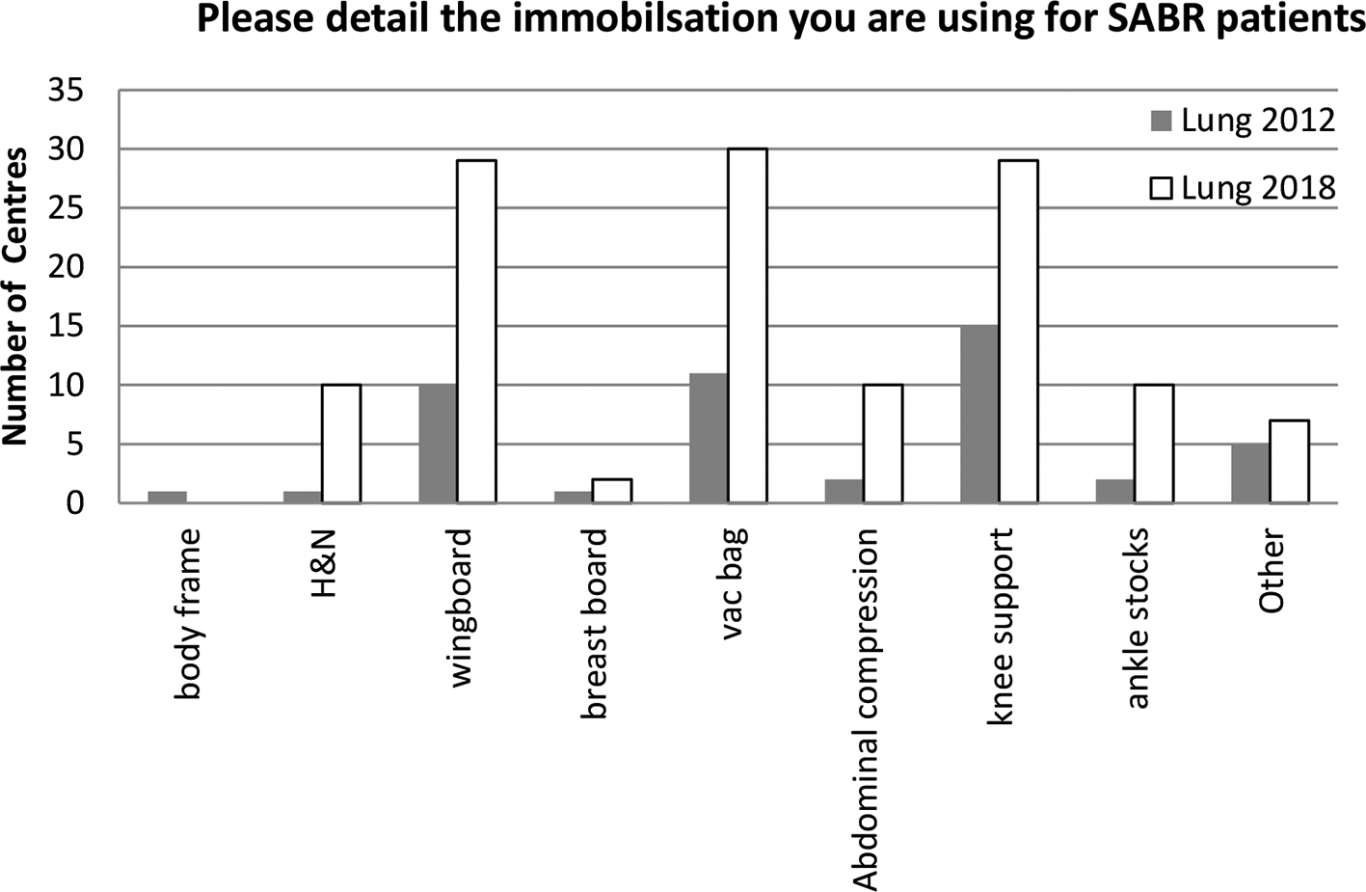
Immobilization devices used in the UK for Lung SABR, comparison with 2012. SABR, stereotactic ablative radiotherapy.

Lung (Figure 3)—the wing board is used by 29/37 (78%) responding centres with several centres using a combination of devices, the most popular combination remains the use of a wingboard with vacuum immobilisation and knee support by 24/37 (65%) of responding centres. There is an increased use of abdominal compression from 2 to 10 centres. "Other" includes thermoplastic shells, BodyFix^®^ (Medical Intelligence, Medizintechnik GmbH, Schwabmunchen, Germany) and mattresses.

Abdominal sites —>50% of centres treating liver, pancreas and adrenal indications have implemented abdominal compression with further centres expressing a future interest.

Amongst 45 responding centres, there is an even split with respect to CT manufacturers with Philips Healthcare (Best, Netherlands), GE Healthcare (Buckinghamshire, UK) and Toshiba (Canon Medical Systems, USA) each in 13 centres and Siemens Healthcare (Erlangen, Germany) in 12 centres (a couple of centres own more than one scanner). All 50 centres are using 4DCT for SABR or intend to use 4DCT once they have a SABR program with a maximum slice thickness of 3 mm. Only one centre is yet to implement 4DCT. 4DCT experience has increased nationally, with 24/35 (69%) responding SABR active centres having treated >100 patients compared to 4/17 (23%) in 2012, Figure 4.

**Figure 4.**
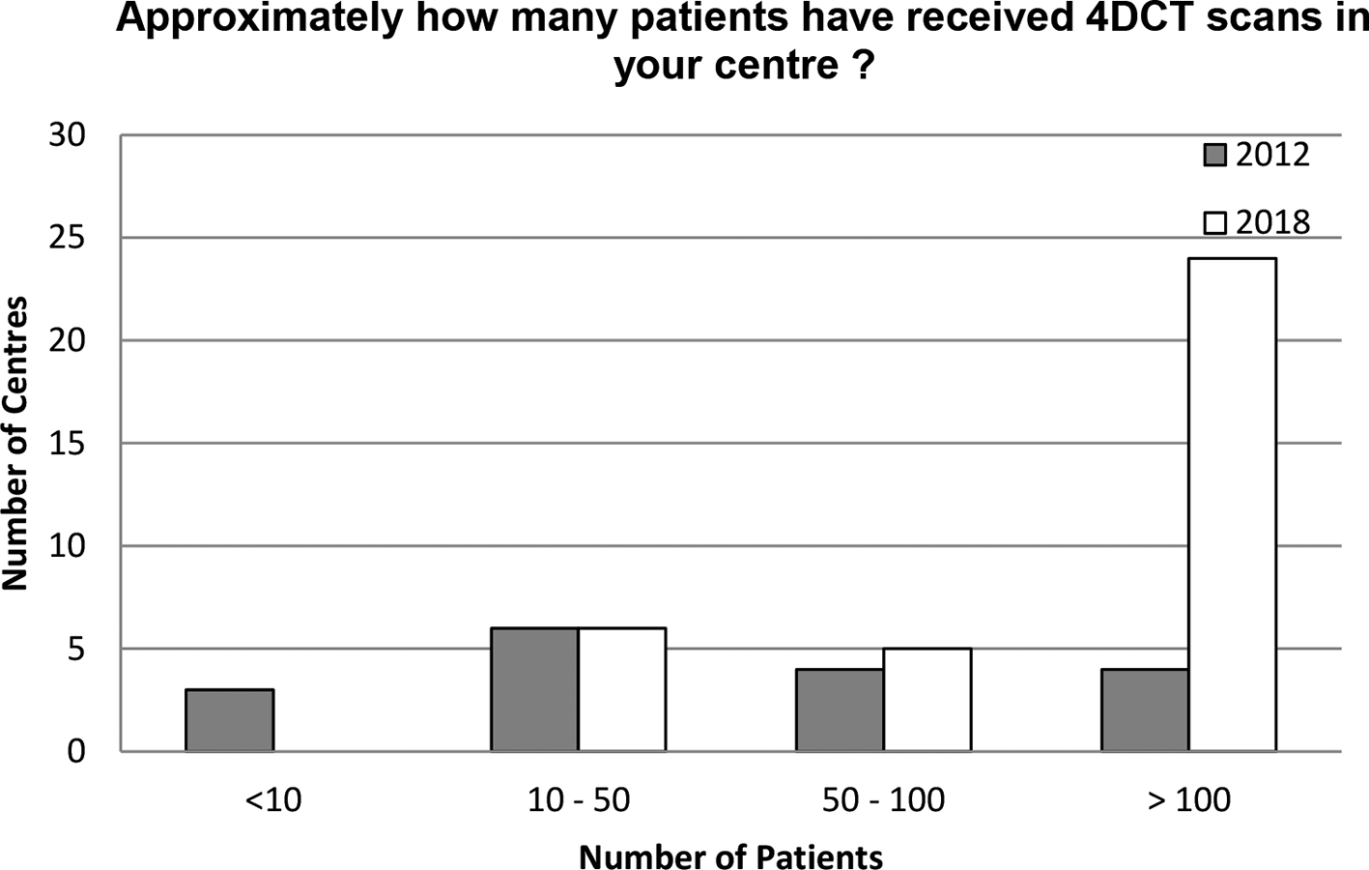
Current provision of 4DCT amongst SABR-active centres in the UK compared to 2012. 4DCT, four-dimensional CT; SABR, stereotactic ablative radiotherapy.

Evaluation of tumour motion prior to 4DCT is no longer easily achievable with the demise of simulators. A couple of centres indicate the use of CBCT or fluoroscopy. Failure rate is low with most responding centres reporting <6% of scans not useable for motion assessment (lung—37/43 and abdomen—18/21) and remains primarily due to issues of patient compliance or breathing irregularity. 4DCT scans are assessed either qualitatively or quantitatively by 42/43 responding centres, with 34/35 (69%) responding SABR active centres making an assessment. Free text responses indicate that scans are still considered acceptable so long as the artefact is not in the region of interest.

**Figure 5.**
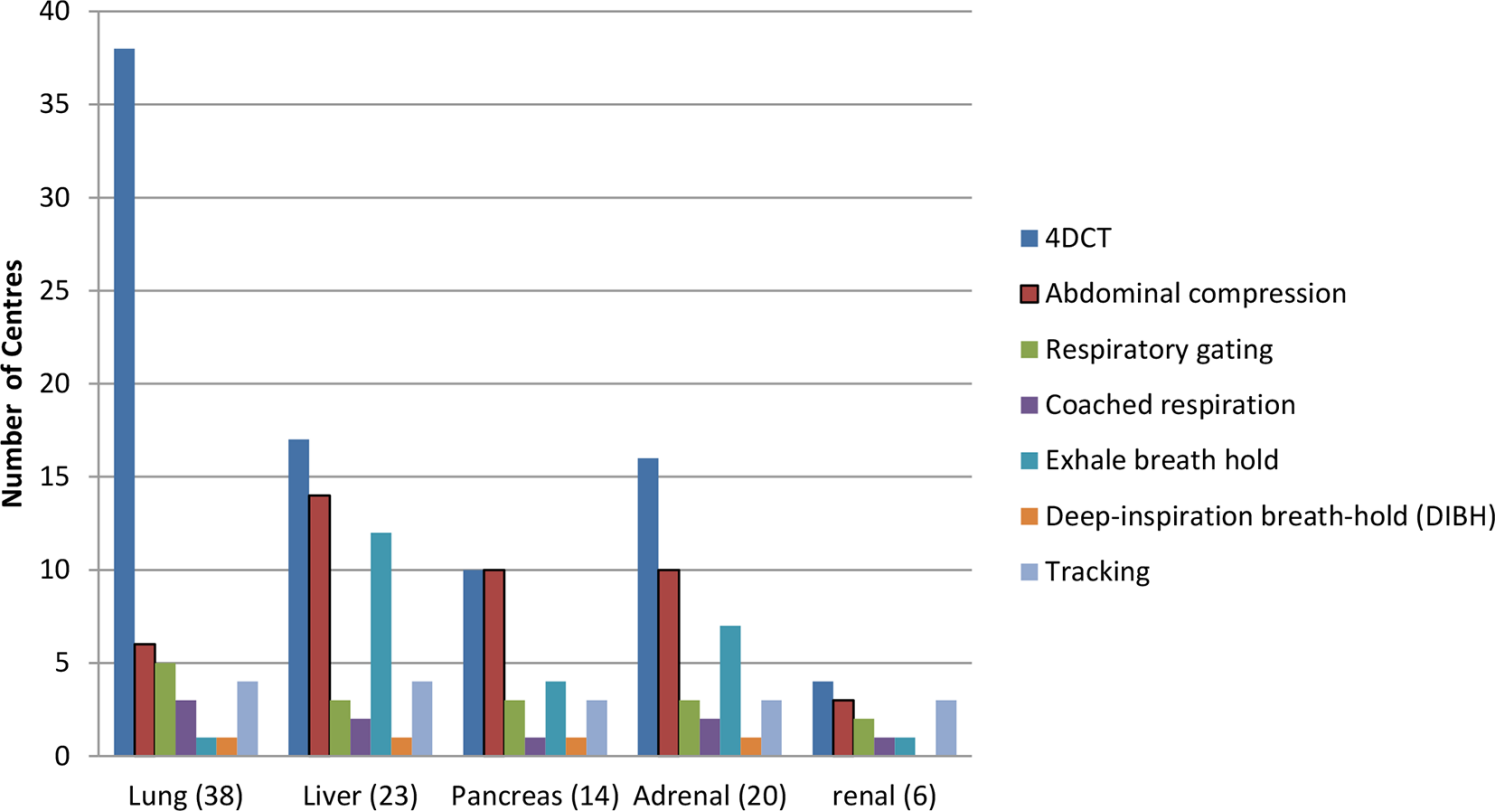
The range of scanning protocols utilised by centres for those treatment sites subject to respiratory motion. 4DCT, four-dimensional CT.

In contrast to 2012, in combination with 4DCT we now see a wide variation of other scanning protocols (Figure 5). This is most likely a consequence of the development of linac-based SABR for treatment sites other than lung. This differs from 2012 when gating, breath-holding and abdominal compression were rarely used in UK SABR practice and is more in line with the US survey of SABR practice^27^ which suggests that 4DCT (used in 75% of centres) is often used in combination with abdominal compression (51%) or gating (31%). For example 12/17 (70%) responding centres indicate the use of 4DCT with abdominal compression for liver.

In lung, there appears to be a preference for imaging the entire scan volume, with 28/46 (61%) of all responding centres and 21/35 (60%) responding SABR active centres using this method. However, in the abdominal region there is an equal split between centres using full/partial scan lengths. Contrast is used by 28/33 (85%) responding centres (14/17 (82%) in 2012) as standard or in selected lung SABR cases. For liver, adrenal and pelvic node SABR, this is the case for >95% of responding centres (19/20, 16/16 and 18/19 respectively). For prostate SABR 9/11 (82%) responding centres do not use contrast, with 2/11 (18%) responding centres using it only in selected patients.

Dose–length product (DLP) values to indicate the typical 4DCT scan dose were reported for 44 scans at 30 responding centres (17 partial and 27 full 4DCTs). For this analysis, partial scan data were excluded. This was due to variation in whether the DLP reported was for just the partial 4DCT scan within the scan protocol or if the reported DLP was for the complete scan protocol and included the three-dimensional CT (3DCT) required for treatment planning. Furthermore, data were not captured on the partial 4DCT scan length which would correlate with DLP.

Lung (Table 3a)—DLP values were found to vary by almost an order of magnitude (range 626–3500). Similar findings were seen in 2012 (range 400–3840) and this also correlates with a recent dose audit completed by IPEM on radiotherapy planning scans.^28^ There is a large dependence on manufacturer, with the Phillip’s and Toshiba scanner tending to yield the lowest DLP and GE the highest. IPEM reported similar findings with Philips and GE scanners, but conversely they found Toshiba yielded the highest DLP along with GE. The difference is most likely due to the very small sample size of Toshiba data in this survey.

**Table 3.** (a) Lung 4D-CT scan DLP values grouped by scanner manufacturer with 2012 values in (). (b) Abdominal 4DCT scan DLP values grouped by scanner manufacturer.

**Manufacturer**	***n***	**DLP mean** (**mGy cm**)	**DLP range** (**mGy cm**)
**(a)**			
Philips Healthcare (Best, Netherlands)	8 (4)	963 (648)	626–1615 (400-800)
GE Healthcare (Buckinghamshire, UK)	6 (5)	2321 (2756)	1000–3500 (1440–3840)
Siemens Healthcare (Erlangen, Germany)	4 (2)	1794 (1550)	800–2936 (1500–1600)
Toshiba (Canon Medical Systems, USA)	2	996	732–1260
**(b)**			
Philips Healthcare (Best, Netherlands)	2	1350	750–1950
GE Healthcare (Buckinghamshire, UK)	3	3717	987–7165
Siemens Healthcare (Erlangen, Germany)	2	1850	1850

4DCT, four-dimensional CT;DLP, dose–length product;IPEM, Institute of Physics and Engineering in Medicine.

Abdominal sites (Table 3b)—5/30 responding centres reported DLP values for 7 full 4DCT scans. Similar to lung these were found to vary by an order of magnitude (range 750–7165) with Philips yielding the lowest DLP.

The manufacturer’s dependence on DLP is likely to do with the different 4D-CT implementation used by different manufacturers, and this is also noted in the IPEM report.^28^ IPEM have proposed a planning scan dose reference level of 1750 mGy.cm for Lung 4D scans. Data were not captured on the use of any dose reduction techniques, and this could also be adding to the large range of values.

### Delineation

Lung—33/35 (94%) responding centres delineate an internal target volume (ITV) to contain respiratory-induced tumour motion within the treated region. There exists a wide variation in the methods used to create an ITV including: directly from the maximum intensity projection or from the union of gross tumour volumes (GTVs) on all or a selection of individual phases. The two centres only delineating a GTV on a selected phase include a CyberKnife^TM^ (Accuray Inc., Sunnyvale, CA) centre and a centre always using abdominal compression or exhale breath-hold. Cyberknife centres use Cyberknife synchrony tracking motion management system and only use a small ITV where the target-surrogate relationship is not perfect for tracked treatments. Other approaches include a permutation of several of the above methods and one centre using the MidVentilation^29^ method.

**Figure 6.**
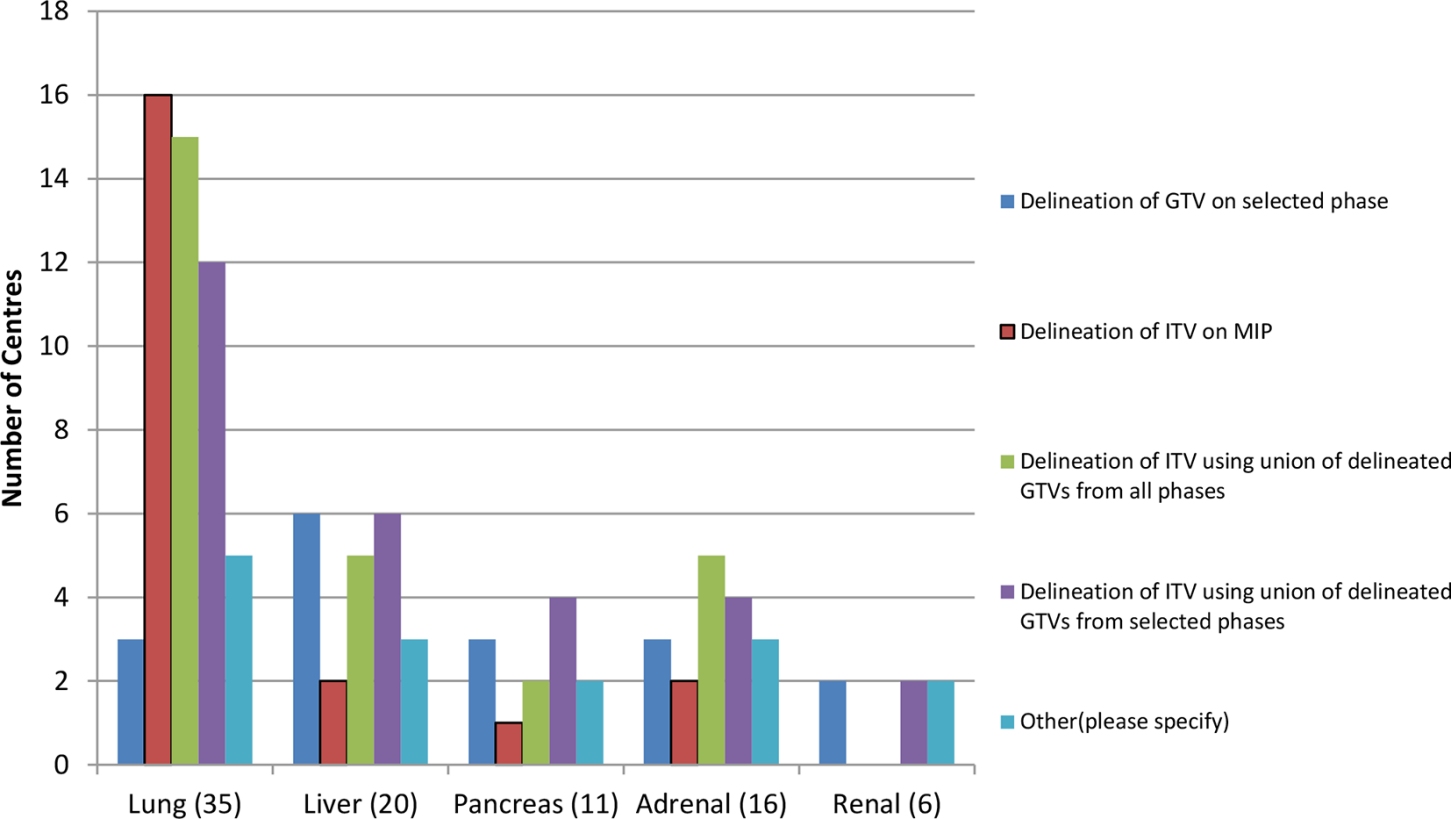
Range of methods used to delineate an ITV. GTV, gross tumour volume; ITV, internal target volume; MIP, maximum intensity projection.

Abdomen—For liver in particular, delineation of a GTV on a selected phase or ITV using a GTV from selection of individual phases is more common (Figure 6). This is most likely due to the increased use of breath-hold techniques and inability to accurately use a maximum intensity projection reconstruction for delineation in this region.

**Figure 7.**
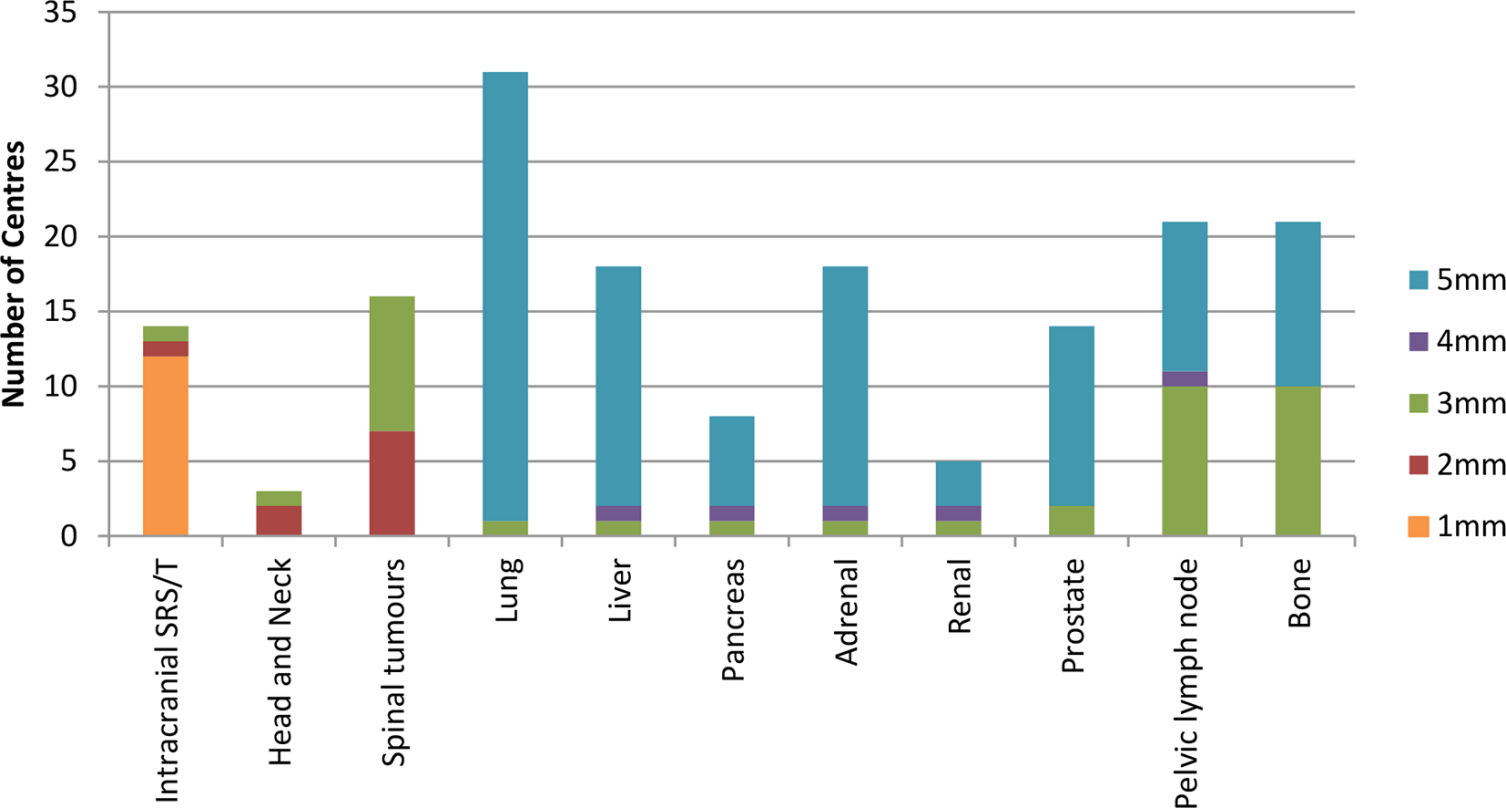
The PTV margin size across a number of treatment sites. PTV, planning target volume.

It is most common to have uniform PTV expansion margins, except in the prostate where a smaller posterior margin is used in line with the PACE trial^18^ and two centres using 6 mm and one centre 7 mm craniocaudally for lung. Reported PTV margins vary from 1 to 6 mm, with 5 mm being more common in treatment sites where there is uncertainty due to breathing motion, *e.g.* lung and liver lesions (Figure 7). This is in agreement with the minimum median CTV-to-PTV margin of 5 mm report in the ESTRO ACROP consensus guidelines.^22^ Three centres said that lymph node margins vary between 3 and 5 mm. Reasons given were tumour type, expected growth, location, motion and visibility of target. One centre reported that for bone SABR patients CTV expansion is variable 0–10 mm based on clinician judgement.

**Figure 8.**
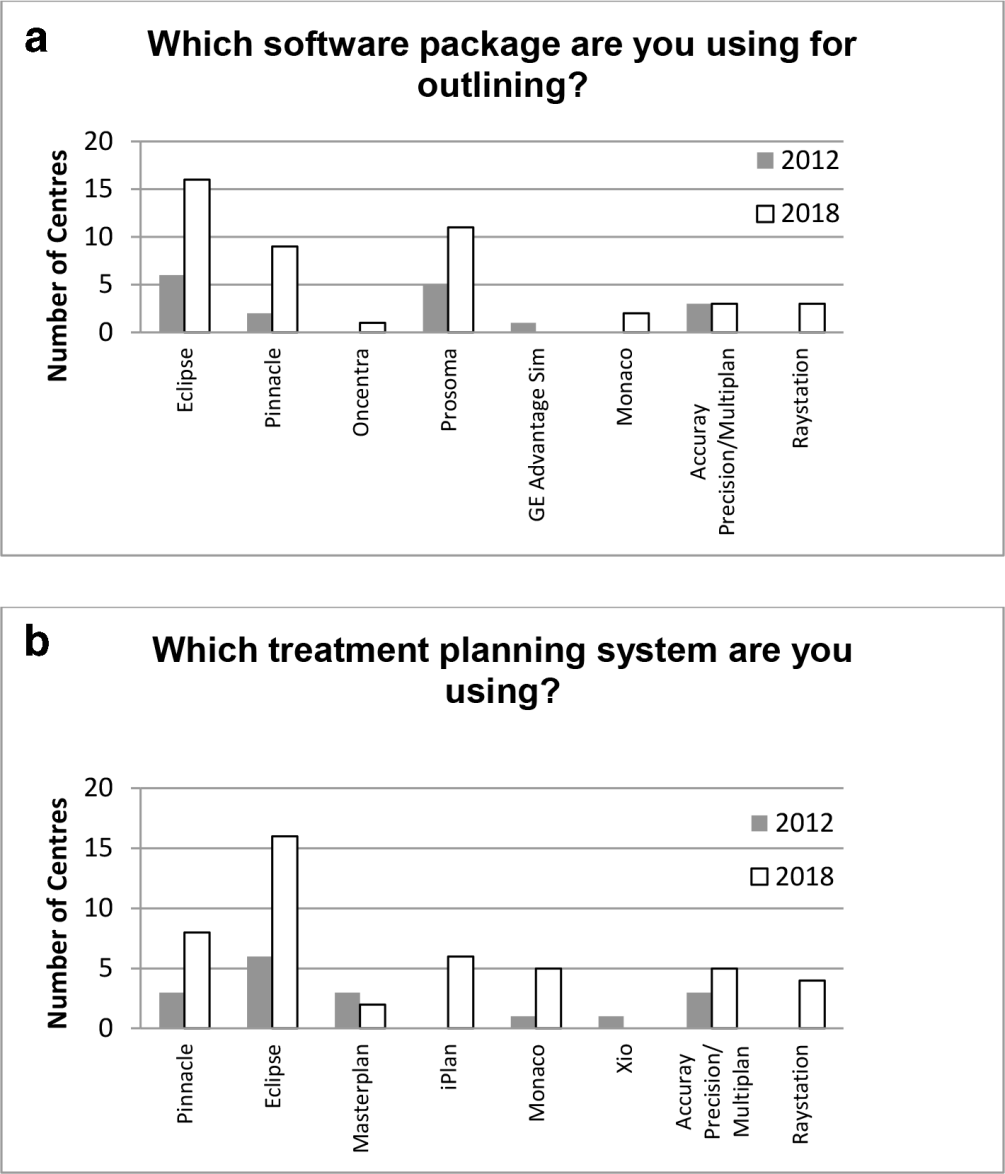
Illustrating the diversity of equipment used throughout the UK to (a) delineate structures and (b) plan SABR treatments. Some centres used multiple systems. Eclipse^™^ (Varian Medical Systems, Palo Alto, CA), GE Adv Sim (GE Health Care, Buckinghamshire, UK), Monaco^®^ (Elekta AB, Stockholm, Sweden), Multiplan^®^ (Accuray Incorporated, Sunnyvale, CA), Pinnacle^®^ (Philips Healthcare, Best, Netherlands), ProSoma (Oncology Systems Limited, Shropshire, UK), Oncentra^®^ Masterplan (Nucletron^™^, Netherlands), Xio (Elekta AB, Stockholm), iPlan^®^ (Brainlab AG, Germany), Raystation (Raysearch Laboratories SB, Sweden). SABR, stereotactic ablative radiotherapy.

Figure 8a and b respectively illustrate the diversity of equipment used throughout the UK to delineate structures and plan lung SABR treatments. 21/36 (58%) responding centres (5/15 (33%) in 2012) said they use some form of auto-segmentation software to aid delineation of organs at risk (OARs mainly lungs, spinal cord and body). Whilst in some centres OARs are drawn by physicists (13), radiographers (14) and dosimetrists (19), free text responses indicated that often contours still require review/sign off by clinicians. In addition, 30/36 (83%) responding centres said that clinicians are still required to delineate more complex OARs. Only 2/36 (6%) responding centres replied yes to radiologist input.

### Treatment planning

For lung, 31/34 (91%) of responding centres are using VMAT compared with 50% in 2012 and all 34 responding centres are using inverse planning (70% in 2012), with only one centre also using forward planning.

At linac centres across all treatment sites, fixed-field IMRT is only used by two centres (but not exclusively) making VMAT by far the most popular delivery method. Five centres are still using non-coplanar beams only if there is an advantage.

All centres are now either using a Type B or Monte Carlo (MC) algorithm for Lung as recommend by national^8^ and European guidelines. However, CyberKnife centres are still using a Type A algorithm for other abdominal SABR sites. In contrast to 2012 when no centres reported the use of MC, there are now nine centres using MC (Accuray (5), Monaco (4)).

### Treatment and verification

**Figure 9.**
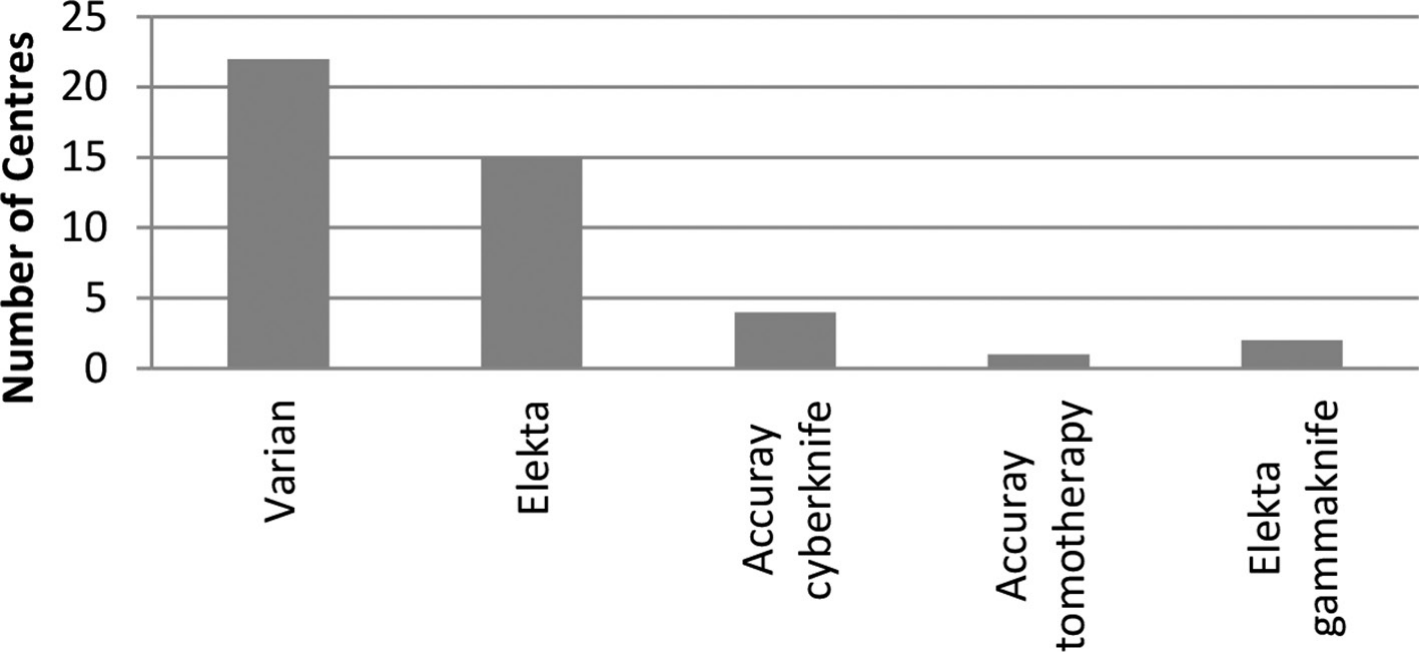
Illustrating the diversity of equipment used throughout the UK to treat SABR. Varian Medical Systems, Palo Alto, CA; Elekta AB, Stockholm; Accuray (Accuray Incorporated, Sunnyvale, CA). SABR, stereotactic ablative radiotherapy.

SABR treatments in the UK are delivered across a variety of platforms (Figure 9). 32/36 responding centres deliver SABR on conventional linear accelerators with CBCT imaging facilities.

An important development over the last 6 years is that SABR for extracranial sites like prostate, pancreas and renal is no longer performed exclusively in Cyberknife centres.

**Figure 10.**
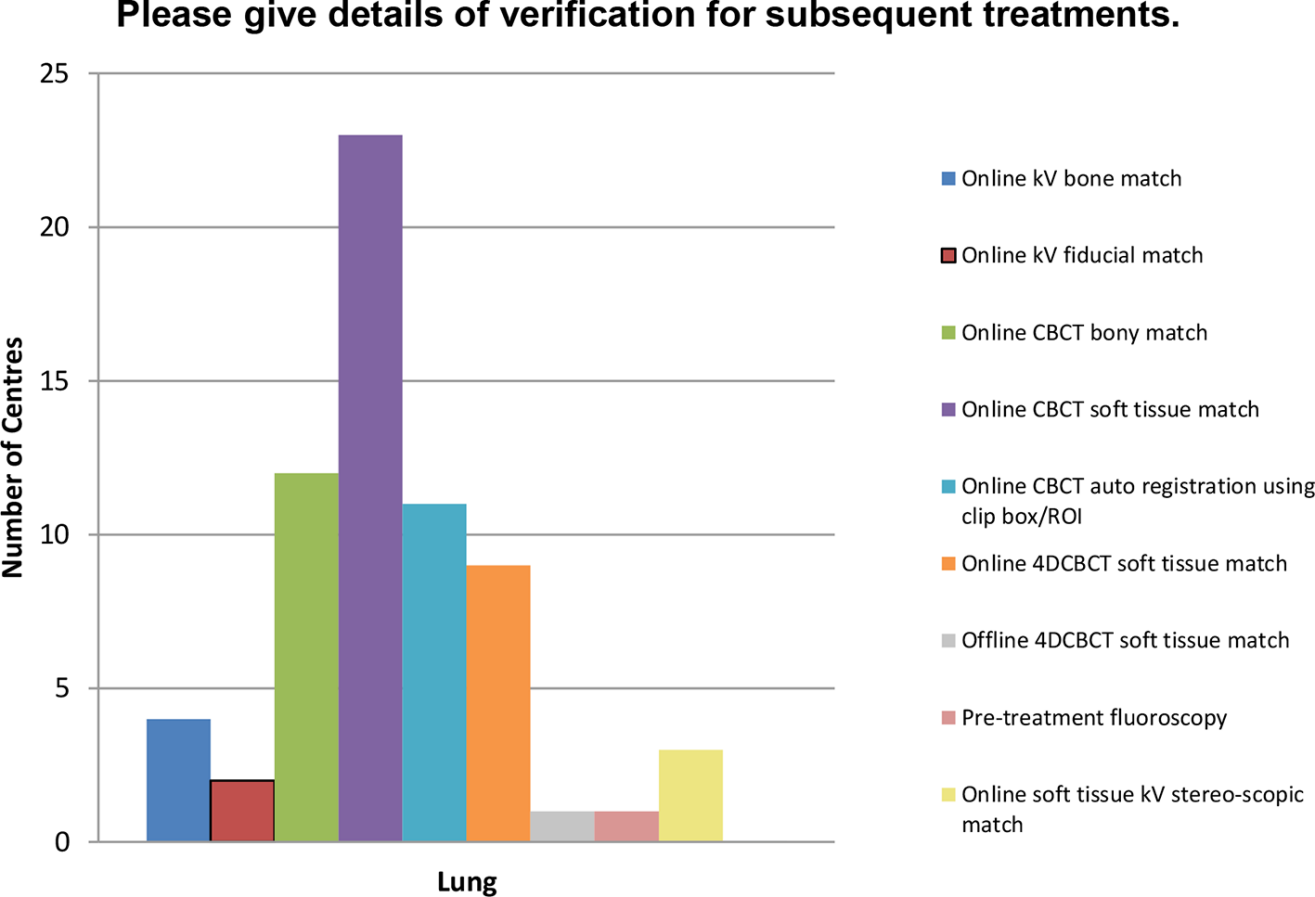
Illustrating range of verification methods used throughout the UK for lung SABR. SABR, stereotactic ablative radiotherapy.

Centres were asked details of their treatment verification at Day0/1 and subsequent fractions. There is a wide range of methods being used as evident in Figure 10 for lung. This picture is similar across other treatment sites. Image guidance is mainly with a soft tissue registration with some centres also using a preliminary automatic match to a region of interest around the PTV. Since 2012 we have seen the introduction of 4DCBT by 10/35 (29%) responding centres for lung. However, only 4 of these 10 centres have chosen to implement 4DCBCT for liver, and fewer for other sites. The use of ExacTrac stereoscopic imaging on the Novalis is reported by six centres for treating spinal tumours. 8/20 (40%) responding centres are using fiducial matching for prostate SABR but only 3/21 (14%) for liver. One centre reports the use of Surface Guided Technology using AlignRT from VisionRT Ltd (London, UK) on all SABR patients

This wide range of verification methods are used in some combination several times per treatment according to each individual department’s protocol. Free text responses indicated that there exists a wide range of workflows among responding centres.

### Quality assurance

The high dose per fraction used in SABR require increased accuracy both in terms of dose calculation and positioning of steep dose gradients, therefore rigorous QA is essential.^8^

20/36 (56%) responding centres perform additional machine QA, mainly involving tighter tolerances and the Winston-Lutz test. Across all treatment sites 35 responding centres said they are still performing patient specific QA. On average across all treatment sites > 50% of these 35 centres perform at least two methods—no change from 2012. For lung, the most frequently used method is Delta4, followed by Arc Check and chamber measurement. All 35 centres said that patient specific QA is performed by a member of the physics staff. In a few centres, other staff are also involved (radiographers 4, technicians 2, dosimetrists 4). Across all treatment sites, a range of acceptance criteria were indicated dependent on equipment used, local or global γ and normalization. However, the most frequently used acceptance criteria was a γ index of 3%/3 mm for two-dimensional and 3D image analysis and dose difference of 3% for chamber measurements.

### Timings for entire process of lung SABR

To help centres allow for the extra time resources required for the implementation of a SABR technique and to allow comparison amongst established centres, the questionnaire asked each centre to state the time required for each major step in the SABR workflow.

The length of 4DCT scan sessions, and planning time was seen to increase (Table 4) since 2012 reflecting increased complexity of cases and perhaps, the planning of multiple lesions for oligometastatic disease. Although lung SABR treatment delivery is on average quicker than for other sites, perhaps since this is the site in which most experience has been gained.

**Table 4.** Summary of the most frequent time at each stage of the SABR process in the UK (^*^quicker if several plans batched together)

	**Mode – 2012** (**min**)	**Mode – 2018** (**min**)
**4DCT**	30–40	50–60
**Target and OAR delineation**	30–60	30–60
**Treatment planning**	>91	>120
Patient-specific plan QA^*^	30–45	15–30
**Daily treatment delivery**	21–30	Lung 21–30 Other 31–40

4DCT, four-dimensional CT;OAR, organ at risk;QA, quality assurance.

In addition, in this survey beam-on time data were captured, with a mode of ≤5 min, indicating that the majority of treatment delivery time is set-up and imaging. This deserves attention both with respect to patient comfort and intrafraction motion. The short beam-on times is most likely a reflection of the documented increased used of VMAT and perhaps increased implementation of FFF (not captured in the Survey).

### Plans for implementation of new techniques

Centres were asked for details of new planning techniques or equipment, new treatment and imaging techniques or equipment that they plan to implement over the next two years. Free text replies included implementation of 4DCBCT (nine centres), 10FFF (seven centres), Raystation (Raysearch Laboratories SB, Sweden) (five centres), Abdominal Compression (four centres), Surface Guided Radiotherapy (four centres), MRI Linac (three centres), Gating (three centres).

## Discussion

As in 2012, this survey has a high response rate (77%) indicating that UK centres remain engaged with SABR and that the survey provides an accurate reflection of current practice and the aspirations of future provision of SABR in the UK. Hence the survey adds robust data for SABR treatment across a range of sites and not just peripheral lung as was the case in 2012. It has been possible to assess how realistic the intentions expressed in 2012 were and note if further investment is still required to increase provision of this clinically advantageous technique. Whilst the number of peripheral lung patients treated per annum has increased (Table 1), it remains below the demand estimated in the NRIG guidance for Commissioners.^9^ This is a concern particularly as Dutch data^30^ suggest that to improve outcome across a population SABR needs to be accessible and delivered at all radiotherapy centres.

The emerging data for treatment of oligometastatic disease from the SABR-COMET^5^ and Gomez trial^31,32^ suggest that there will be a role for SABR going forward. The UK has an important role in confirming these finding through our current portfolio of studies^16–18^ and it is worrying that a number of centres report significant challenges in contributing to these studies. Increasing the support for radiotherapy trials needs to be a priority for the NHS to ensure these important studies are completed so that we have an evidence base that can lead to commissioning of an appropriate level of SABR treatment for oligometatstic disease once the current NHS program^14^is completed.

The other challenge is the rapid development and adoption of innovative approaches (*e.g.* MR Linac) that are likely to impact of the delivery of SABR treatment over the next 5–10 years. These changes will require us to streamline our SABR education and training and allow the necessary complex quality assurance through the collaborative approach adopted by the UK SABR Consortium and seen in the Australia and New Zealand SABR service.^24^

The survey also provides reassurance that SABR treatments in the UK are largely performed according to UK SABR Consortium Guidelines^8^ and European recommendations/guidelines.^22,25^ This is especially true with respect to immobilization and the predominant use of 4DCT (mandated by ESTRO ACROP^22^) with a maximum slice thickness of 3 mm.

The recommended use of contrast by the Consortium^8^ and AAPM^23^ guidelines for the hepatic system is followed for liver and adrenal sites by 19/20 and 16/16 responding centres respectively. ESTRO ACROP consensus guidelines^22^ report that for lung the ITV concept for delineation as a mandatory minimum and MidV as recommended and VMAT recommended for best SBRT practice. Again, compliance is high with 94% (33/35) and 91% (31/34) of responding centres using an ITV and VMAT approach respectively. Results indicate that centres are also following advice in limiting non-coplanar beams together with increased use of VMAT and flattening filter free (FFF) to keep treatment times as short as possible and in so doing reducing intrafraction motion.

Documented changes in practice since 2012 include the development of Linac delivered SABR to most non-lung sites, notable increase in number of centres using abdominal compression as part of the immobilisation process (14 *vs* 2 centres) and the introduction of 4DCBCT in the imaging verification process. Of the 10 centres using 4DCBCT, 8 are Elekta sites, which is most likely a reflection of Varian’s 4DCBCT solution until recently not being well integrated into an online workflow. EORTC recommend that 4DCBCT is preferable over 3DCBCT, and therefore the intention of 9 more centres to implement this technology is welcome. A wide range of approaches were noted in accounting for tumour motion, target ITV delineation and treatment image verification. Comparable results focussing specifically on Lung SABR image-guided radiotherapy were recently published by the Cancer Research UK Advanced Radiotherapy Technologies Network (ARTNET) .^33^

The fact that there appears to have been no notable reduction in patient specific QA is not surprising given the extensive use of VMAT. In the ESTRO ACROP guidelines 50% of institutions consider patient–individual QA of VMAT planning mandatory and 50% as recommended.^22^ However, it is worth noting that the time spent per patient on QA is dropping suggesting improvements in process. An increase in time spent at some stages of the SABR process was also seen which is likely to be a reflection on the increased complexity of cases treated.

## Conclusion

This 2018 survey shows a welcome increase in SABR provision across the UK, surpassing projections in 2012. However, with emerging data it is clear that the UK SABR program needs to continue its expansion to ensure that patients with oligometastatic disease have access and SABR for early stage lung is deliverable in all centres. Implementation of novel technology is noted, however, guidance to address variability in target delineation, image guidance and possible reduction in patient specific QA is warranted.
